# Syphilis Maligna Præcox and Its Treatment

**Published:** 1909-02-06

**Authors:** 


### Medicine.

### SYPHILIS MALIGNA PRÆCOX AND ITS TREATMENT.

Every now and then a case of very severe syphilis is met with, owing to exceptional virulence of the infection or lack of resistance in the patient; and there is one type of the disease in which there is no roseola, mucous plaques are absent, and yet the skin lesions are extremely severe—ulcerating even in the early months when most cases would be described as in the secondary stage. Certain suggestions made by M. Louis Queyrat* are very much to the point, and they will be considered presently; but first the notes of two of his illustrative cases may be given to indicate precisely what is meant by syphilis maligna præcox:—

### Two Illustrative Cases.

The first case is that of a man aged thirty, who developed a chancre one February. The diagnosis was made at a hospital. In March, but not so suddenly that the exact date could be specified, an eruption of small red spots appeared; each spot projected slightly above the surface of the skin, and at the very centre it became pustular. By May the man was excessively ill. His body was covered with lesions, some of which were vesicular, some pustular, some ulcerating, some covered with prominent crusts upon an ulcerating base. There were 54 such lesions upon the trunk, 18 upon the right leg, 11 upon the left, 8 upon the right arm, 9 upon the left, 1 in the right submaxillary region, 1 just below the lower lip, 1 between the eyebrows, 1 upon the forehead, and 4 upon the hairy scalp. There was no lesion upon either palm, nor upon the sole of either foot.

The lesions presented different appearances, according to their age, and they varied greatly in size; the smallest being circular and no bigger than lentils, whilst the largest were oval, measuring an inch and a half in length by an inch in breadth. Each lesion began as a small round plaque of a dull red colour; upon the centre of this erythematous plaque the epidermis next began to be raised. Serous exudation then occurred, and the contents of the vesicle rapidly became turbid; the vesicle or vesico-pustule then broke, or else dried without breaking, and a crust formed, under which the rest of the epidermis ulcerated until the dermis was exposed. If the crust was raised up, a smooth-bottomed flat ulcer was exhibited, with clean cut edges and a thin surrounding rim of red. The surface of each ulcer kept on discharging a secretion which solidified into fresh crusts of a brownish black colour, more or less stratified, and assuming shapes characteristic of ecthyma or rupia.

Although the skin was so extensively affected, there was no lesion of any mucous surface. The patient was in agony all the time from the pain of the deep ulcers; he could not rest quietly in any position in bed, and he was constantly crying out. He became pale and depressed, lost his appetite, and was altogether very ill. Night after night he was unable to sleep at all. There seemed to be no particular reason why the disease should have been so virulent in this man. He was well-built, of moderate stature; he was not addicted to drink, and hitherto he had always been strong and well.

Another case exhibits the great tendency of this form of the disease to relapse, notwithstanding every effort that is made to effect a complete cure. The patient was a man aged 26, who had had an ordinary chancre in June 1906, and three weeks afterwards developed syphilis maligna præcox similar to that described in the first case. Since then there had been repeated recurrences of the ulcerative and crusty skin lesions, very little benefited by mercury and iodides.

Two years after the chancre there were ulcers and rupial crusts upon the trunk, buttocks, elbow, shoulder, neck, and hairy scalp, together with cicatrices scattered over different parts. Some of these scars were obviously recent and of a dull red colour, others were older and colourless. Both kinds were the results of former exacerbations in the cutaneous syphilides.

### Treatment.

When one pays particular regard to cases of the above type one notices that they exhibit no roseola and no mucous plaques. Moreover, mercurial treatment gives little relief, iodides perhaps a little more, but not constantly. The second of the above cases was treated at first by calomel, mercurial injections, and iodides. The lesions only began to improve at the end of four months, and then but to a slight degree. A month later, in November 1906, there was a relapse. Treatment by mercurial inunctions and by iodides brought about little improvement, nor did any of the various methods of mercurial treatment that were employed from time to time seem to do any good. Five serious exacerbations of the rupio-ulcerative syphilides occurred within two years.

Atoxyl, which has done good in some instances, failed too. Dr. Queyrat gives it as his opinion that in cases of this kind it is better not to persist with any treatment by mercurials, by iodides, or by atoxyl, but rather to treat the patient on more general lines. He recommends good food and plenty of it, including abundance of proteid; injections of cacodylate (three-quarters of a grain) alternately with injections of sea water (one and a half ounces); syrup of the iodide of iron by the mouth, and, especially in winter, cod-liver oil; whilst local treatment to the sores needs to be adopted at the same time, hydrogen peroxide lotion of suitable strength sometimes being of great use here.

Treatment carried out upon such lines as we have indicated will sometimes relieve the patient more effectually and quickly than will any of the drugs used in ordinary cases. This was shown by Dr. Queyrat's first case. The condition was lamentable when this man first came under observation. He was treated precisely upon the lines just mentioned,

* "Bulls. et Mem. de la Soc. Med. des Hôp. de Paris," 1908, p. 147.

and in two months his weight increased by nearly half a stone, and the ulcerating and encrusted sores entirely healed, leaving only purplish-red marks and scars where they had been.

Not only is this peculiar type of syphilis one of extreme severity as far as the skin is concerned, though free from roseola, free from mucous plaques, and very resistant to mercurials and iodides; but also, when the cases are followed up it would seem that they are peculiar in not being liable to visceral complications nor to lesions of the nervous system. Their lesions are those of the integuments, to which may be added ulcerations of the soft palate, of the nasal mucous membrane, or of the mouth.

